# The use of hyperbaric oxygen for veterans with PTSD: basic physiology and current available clinical data

**DOI:** 10.3389/fnins.2023.1259473

**Published:** 2023-10-25

**Authors:** Keren Doenyas-Barak, Ilan Kutz, Erez Lang, Rachel Merzbach, Rachel Lev Wiesel, Rahav Boussi-Gross, Shai Efrati

**Affiliations:** ^1^Sagol Center for Hyperbaric Medicine and Research, Shamir Medical Center, Zerifin, Israel; ^2^School of Medicine, Tel Aviv University, Tel Aviv, Israel; ^3^The Louis and Gabi Weisfeld School of Social Work, Bar-Ilan University, Ramat Gan, Israel; ^4^The Emili Sagol Creative Arts Therapies Research Center, University of Haifa, Haifa, Israel

**Keywords:** post-traumatic stress disorder, hyperbaric oxygen therapy, treatment-resistant PTSD, combat-associated PTSD, neuroplasticity

## Abstract

Post-traumatic stress disorder (PTSD) affects up to 30% of veterans returning from the combat zone. Unfortunately, a substantial proportion of them do not remit with the current available treatments and thus continue to experience long-term social, behavioral, and occupational dysfunction. Accumulating data implies that the long-standing unremitting symptoms are related to changes in brain activity and structure, mainly disruption in the frontolimbic circuit. Hence, repair of brain structure and restoration of function could be a potential aim of effective treatment. Hyperbaric oxygen therapy (HBOT) has been effective in treating disruptions of brain structure and functions such as stroke, traumatic brain injury, and fibromyalgia even years after the acute insult. These favorable HBOT brain effects may be related to recent protocols that emphasize frequent fluctuations in oxygen concentrations, which in turn contribute to gene expression alterations and metabolic changes that induce neuronal stem cell proliferation, mitochondrial multiplication, angiogenesis, and regulation of the inflammatory cascade. Recently, clinical findings have also demonstrated the beneficial effect of HBOT on veterans with treatment-resistant PTSD. Moderation of intrusive symptoms, avoidance, mood and cognitive symptoms, and hyperarousal were correlated with improved brain function and with diffusion tensor imaging-defined structural changes. This article reviews the current data on the regenerative biological effects of HBOT, and the ongoing research of its use for veterans with PTSD.

## Introduction

Epidemiological studies have consistently revealed high prevalences of combat-associated post-traumatic stress disorder (PTSD) among military personnel and veterans, affecting up to 30% of those with a history of combat involvement (Marmar et al., 2015). Rates of PTSD vary, depending on the specific conflict, as well as the duration and intensity of combat exposure (Hoge et al., 2008; Jankowsi, 2016). Furthermore, increased risks have been reported among persons with physical injuries (Hoge et al., 2008). Combat-associated PTSD substantially impacts not only the mental and physical health of individuals but also their social functioning and overall quality of life.

The primary treatment options for PTSD typically involve psychological therapies and pharmacotherapy. Most guidelines recommend trauma-focused cognitive-behavioral therapy or pharmacotherapy based on the clients’ preferences (Martin et al., 2021). However, numerous studies have demonstrated only marginal superiority of these treatments compared to control conditions (Steenkamp et al., 2015), and also low tolerability and high dropout rates (Hembree et al., 2003; Imel et al., 2013). Moreover, real-world clinical settings have shown even lower effectiveness and response rates (Nathan et al., 2000; Hengartner, 2018). To gain insights into the effectiveness of PTSD therapies in real-life situations, the Israel Defense Forces Unit for Treatment of Combat-Related PTSD has collected data on treatment outcomes. A retrospective analysis of 709 veterans seeking treatment revealed that only 39% experienced significant clinical improvement; and the rate of remission for intrusion symptoms was only 16% (Levi et al., 2022). Together with other studies, this suggests that combat veterans present particular challenges in treatment response (Van der Kolk, 1994).

Treatment resistance in veterans with PTSD may be explained by functional and structural brain changes, as evidenced by imaging studies, particularly within the frontolimbic circuitry (Yehuda et al., 2015). Such brain impairments underscore the potential benefit of biological treatments.

In 2018, the VA Evidence-Based Synthesis Program for traumatic brain injury (TBI) and/or PTSD stated that based on the data available up to 2018, it was difficult to make clear decisions regarding the use of HBOT for TBI and PTSD (Peterson et al., 2018). However, since 2018 preclinical as well as clinical data accumulated (Deru et al., 2018; Lin et al., 2019; Lippert and Borlongan, 2019; Mozayeni et al., 2019; Harch et al., 2020; Doenyas-Barak et al., 2022, 2023; Hadanny et al., 2022; MacLaughlin et al., 2023) and contributed to our understanding of the potential role of HBOT in PTSD treatment.

## Pathophysiology and long-term consequences of trauma

Accumulating evidence suggests that the pathophysiological changes occurring during an acute traumatic event can lead to long-term alterations in the structure and function of the brain. The neurobiological cascade begins with an unbalanced surge of stress hormones, characterized by a low ratio of cortisol to catecholamine levels (Yehuda, 2002; Charney, 2004; Pitman et al., 2012). Notable in the early stages following the traumatic event changes in brain perfusion and metabolism can be observed (Lucey et al., 1997; Bonne et al., 2003; Zhe et al., 2016; Ben-Zion et al., 2020). Subsequent long-term changes primarily affect the prefrontal cortex and the limbic system (Yehuda et al., 2015) and correlate with reported clinical symptoms.

Studies have identified abnormalities in the medial prefrontal cortex and the anterior cingulate cortex. Both these regions, when impaired, have been associated with deficits in emotional regulation and can serve as predictors of post-traumatic symptom severity (Zhe et al., 2016). Furthermore, impaired connectivity, both functional and structural, between the amygdala, the hippocampus, and the frontal lobes have been demonstrated (Mueller et al., 2015). These findings support the notion that dysfunction within the frontolimbic circuitry contributes to the difficulty experienced by individuals with PTSD in integrating cognitive control over the emotional neural system.

In addition to alterations in brain metabolism and activity, traumatic exposure has been linked to reduced hippocampal volume, while preserved hippocampal volume has been associated with better response to certain treatments (Admon et al., 2013).

## Hyperbaric oxygen therapy

Hyperbaric oxygen therapy (HBOT) involves the inhalation of 100% oxygen at pressures exceeding 1 atmosphere absolute (ATA). This enhances the amount of oxygen dissolved in the plasma and subsequently the body tissues (Fosen and Thom, 2014). In the blood, oxygen is carried in two forms: a fraction that is bound to hemoglobin and a free fraction dissolved in the plasma. At physiologic normoxic conditions, i.e., at a normal concentration of inspired oxygen (20.8%), at 1 ATA, up to 99% of the oxygen is carried by hemoglobin, while the fraction of oxygen dissolved in the plasma is small (Collins et al., 2015). However, according to Henry’s law (Trayhurn, 2019), at an elevated pressure (such as breathing pure oxygen under hyperbaric exposure), the dissolved amount can become significant. At the cellular level, the oxygen pressure delivered to the mitochondria at 1 ATA is 1–4 mmHg; while in a hyperbaric environment of 2 ATA with 100% oxygen, the pressure may increase by as much as 15-fold (Hadanny and Efrati, 2020). While many beneficial effects of HBOT can be attributed to the steep rise in tissue oxygenation, it is now understood that the fluctuation in the combined action of hyperoxia and hyperbaric pressure triggers both oxygen and pressure-sensitive mechanisms.

One of the most powerful inducers of regenerative processes is low oxygen, or hypoxia (Steenkamp et al., 2015). Interestingly, at the cellular level, it is fluctuations of oxygen concentration and not the absolute values that are sensed. The implication is that by repeating intermittent fluctuations of oxygen, from high oxygen pressures back to normal pressures, the cellular response is that of “relative hypoxia” which in turn, induces the regenerative effects of hypoxia. This is described as the “hyperoxic-hypoxic paradox” (Hadanny and Efrati, 2020). This paradox induces a number of physiological effects. These include: improved mitochondrial function, multiplication, and migration; induction of the hypoxic induced factor (HIF); neuronal stem cell proliferation; production of vascular endothelial growth factor (VEGF); and an anti-inflammatory effect. Each of these is described below.

### Improved mitochondrial function, multiplication, and migration

At the cellular level, 80% of the available oxygen is used by the mitochondria. The low oxygen level in this organelle renders it a key oxygen sensor and an important signaler (Palmeira et al., 2019). The effects of HBOT on mitochondrial function and multiplication were demonstrated in several studies. In a training mice model, HBOT facilitated mitochondrial oxidative and glycolytic capacities and increased the expression of proteins involved in mitochondrial biogenesis (Suzuki, 2017). Similar effects were demonstrated among middle-aged athletes who were treated by HBOT; the mitochondrial mass and the related maximal oxygen phosphorylation capacity increased (Hadanny et al., 2022). Other studies highlights the importance of mitochondrial function for proper maintenance of neuronal function. One of the established mechanisms is related to cell–cell signaling via the transfer of mitochondria between astrocytes and neurons (Davis et al., 2014; Hayakawa et al., 2016). Neurons can release and transfer damaged mitochondria to astrocytes for disposal and recycling (Davis et al., 2014), and astrocytes can release functional mitochondria that enter into neurons (Hayakawa et al., 2016). HBOT facilitates these mechanisms, and contributes to neuron resilience to inflammatory insults (Palzur et al., 2008; Lippert and Borlongan, 2019) and to recovery at the chronic delayed stage of various types of brain injuries (Palzur et al., 2008; Lippert and Borlongan, 2019).

### Hypoxic induced factor

Hypoxic induced factor is a transcription factor that responds to changes in cellular oxygen supply (Hellwig-Bürgel et al., 2005). In normoxic conditions, HIF is degraded by hydroxylation, in a process that is regulated by the ratio of reactive oxygen species (ROS) to scavenging activity. During hyperoxia, increased oxygen availability enhances ROS production, but also the production of ROS scavengers, including glutathione peroxidase and superoxide dismutase. Upon return to normoxia, the level of scavengers is increased, according to their inherent elimination half-life, which is significantly longer than the ROS half-life. This results in a low ratio of ROS/scavenging capacity, a state similar to that of the hypoxic state, with increased HIF expression due to suppressed HIF hydroxylation. The effect of repeated intermittent hyperoxia by HBOT on HIF expression was demonstrated in a number of animal models, and in various types of organs and cells (Salhanick et al., 2006; Ren et al., 2008; Hu et al., 2014). Increased HIF expression is neuroprotective and enhances regenerative effects in post-stroke and spinal cord injuries (Angels Font et al., 2010). HIF activation was shown to have a direct effect on hippocampal activity and on hippocampal based memory performance (Adamcio et al., 2010; Xing and Lu, 2016).

### Neuronal stem cell proliferation

Hyperbaric oxygen therapy has been shown to induce the proliferation and mobilization of hematopoietic and mesenchymal stem cells (Milovanova et al., 2009; Thom et al., 2011; Heyboer et al., 2014; Yang et al., 2017), as well as of neuronal stem cells in the hippocampus and the periventricular region (Wang et al., 2007; Yang et al., 2008; Zhang et al., 2010). This effect contributes to regeneration following stroke, TBI, and vascular dementia (Zhang et al., 2010; Lee et al., 2013; Yang et al., 2017), and is facilitated by the elevation of stem cell factors that promote stem cell proliferation (Thom et al., 2006) and stabilization of cAMP responsive element binding protein (Mu et al., 2013).

### Vascular endothelial growth factor production

Vascular endothelial growth factor production is triggered by HIF-1, and in turn activates vascular cells to initiate angiogenesis and arteriogenesis. Angiogenesis is the budding of new capillaries from existing vessels. Arteriogenesis is the remodeling of collateral blood vessels that handle the increased flow, bypassing stenotic regions of the original conduit arteries (Van Weel et al., 2008; Semenza, 2011). VEGF also induces vasodilatation activity, as well as microvascular permeability, which is needed for immediate improvement of tissue ischemia (Semenza, 2011). VEGF was shown to contribute to improved hippocampal activity and neurogenesis (Hassouna et al., 2016).

### Anti-inflammatory effect

Hyperbaric oxygen therapy reduces inflammatory reactions (Vlodavsky et al., 2006), attenuates microgliosis and astrogliosis reactions (Lim et al., 2013; Lavrnja et al., 2015), and promotes blood–brain barrier integrity.

The cellular mechanisms mentioned above contribute to improved cerebral integrity and plasticity. Examples include regeneration of axonal white matter, angiogenesis, improvement in global cerebral blood flow, and increased brain metabolism. Accordingly, HBOT has been shown to improve the maturation, myelination (Haapaniemi et al., 1998; Vilela et al., 2008; Chang et al., 2009), and stimulation of axonal growth, thus enhancing the functioning and communication of neurons (Mukoyama et al., 1975; Bradshaw et al., 1996). In addition, HBOT initiates and facilitates angiogenesis, which also contributes to axonal regeneration (Kuffler, 2011; Lin et al., 2012; Duan et al., 2014; Peng et al., 2014). HBOT improves regional cerebral vascular flow, which is necessary for neurogenesis and synaptogenesis (Chen et al., 2003; Jiang et al., 2005; Tal et al., 2015). In addition to increased regional cerebral blood flow by angiogenesis, HBOT improves global cerebral vascular flow (Neubauer and James, 1998; Rockswold et al., 2001, 2007; Zhou et al., 2007). Due to increased blood flow and oxygenation, brain metabolism increases significantly, as seen in positron emission tomography (PET) and single photon emission computed tomography (SPECT) scans (Boussi-Gross et al., 2013).

**FIGURE 1:**
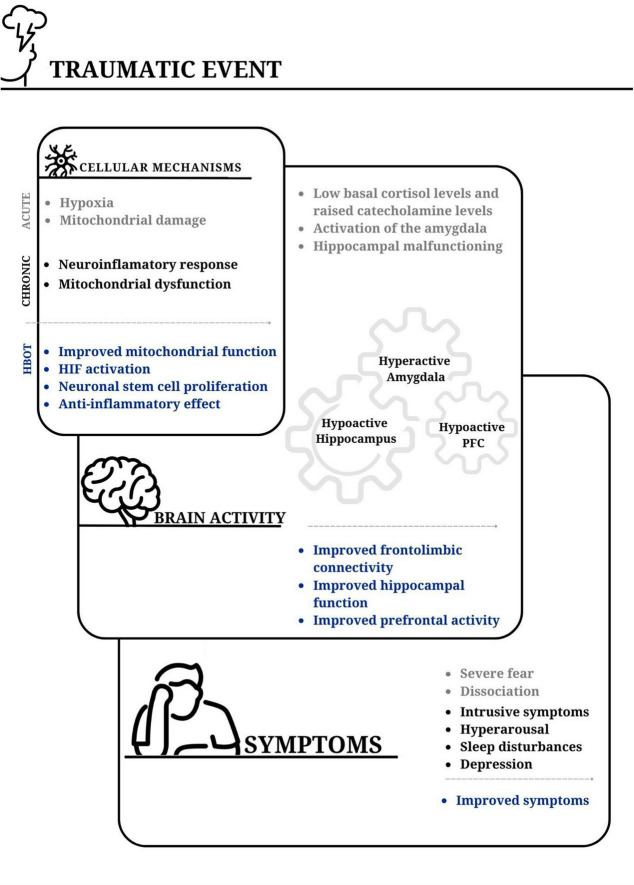
The impact of traumatic events on cellular mechanisms, brain activity, and PTSD symptoms; and the potential benefits of hyperbaric oxygen therapy. Acute stress-inducing events can lead to cellular hypoxia, and widespread mitochondrial damage. The acute event is followed by a neuroinflammatory response and long-lasting mitochondrial dysfunction. An imbalance is commonly observed between the surge of catecholamines and cortisol levels. This contributes to activation of the basolateral amygdala and reduced perfusion to the hippocampus. Subsequently, the hyperactive amygdala, together with reduced activity in the prefrontal cortex and hippocampus, in addition to diminished frontolimbic connectivity, contribute to difficulties in integrating traumatic memories. From a clinical perspective, events that trigger post-traumatic symptoms result in a perceived overwhelming threat and often peritraumatic amnesia, which is presumably associated with hippocampal malfunction. The incomplete acquisition of traumatic memories may contribute to their intrusive nature at a later time. Hyperbaric oxygen therapy (HBOT) has been shown to enhance mitochondrial function and signaling. Additionally, fluctuations in cellular oxygen levels lead to increased hypoxia-inducible factor (HIF) levels. These, in turn, contribute to the activation of genes involved in the repair process. This therapy also promotes stem cell proliferation in various tissues, including neuronal stem cells in the brain. The enhanced activity and connectivity of the prefrontal cortex play a role in achieving better frontolimbic balance. This potentially explains the improvement in hyperarousal symptoms. Furthermore, the improved function of the hippocampus may facilitate the retrieval of inaccessible memories, aid in the processing of traumatic memories, and reduce intrusive symptoms. PFC, prefrontal cortex.

Long-lasting hibernating brain regions are commonly demonstrated in various types of brain injury, such as following concussion and stroke. HBOT targets the baseline pathophysiology that is responsible for unrecovered brain tissue (Efrati and Ben-Jacob, 2014), and induces cerebral plasticity and repair of chronically impaired brain functions. This improves the quality of life of individuals after stroke or prolonged post-concussion syndrome (PCS), even years after the acute event (Boussi-Gross et al., 2013; Efrati et al., 2013; Tal et al., 2015; Yan et al., 2015; Harch et al., 2017; Hadanny et al., 2018a; see Figure 1).

## An evidence-based review of the use of HBOT for veterans with PTSD

The effect of HBOT on the post-traumatic response has been studied extensively in preclinical and clinical trials (Table 1). Several preclinical studies using animal models have demonstrated the salutary effects of HBOT on anxiety-related behavior, neuronal plasticity, neurogenesis, and angiogenesis (Peng et al., 2010; Lin et al., 2019; Fedida et al., in press). HBOT was shown to increase the expression of brain-derived neurotrophic factor and laminin, markers associated with neuronal plasticity and improved dendrite morphology in the hippocampus. HBOT also attenuated the fear response and anxiety-like behavior induced by traumatic stress exposure.

**TABLE 1:** Summary of clinical trials.

References	*N* with PTSD/*N* total	TBI comorbidity	Military	ATA	Number of sessions	Session length (min)	Placebo	Imaging	Outcome
Doenyas-Barak et al., 2022 (controlled trial)	35/35	None	+	2 ATA	60	90	NA	MRI	Improved post-traumatic symptoms (CAPS)
Harch et al., 2020 (controlled single blind)	?/63	100%	No	1.5 ATA	40	60	NA	None	Improved post-traumatic symptoms (PCL), improved sleep quality
Mozayeni et al., 2019	7	100%	Some	1.5 ATA	40–80	45	NA	None	Improved ANAMT4 mood score
Hadanny et al., 2018b	30	None	None	2 ATA	60	90	NA	SPECT and MRI	Improved PTSD symptoms (PSS-I)
Deru et al., 2018 (randomized controlled trial, BIMA study)	18	100%	+	1.5 ATA	40	60	1.2 ATA	None	Improved post-traumatic symptoms (PCL)
Harch et al., 2017 (case-control study)	10/29	100%	+	1.5 ATA	40	60	NA	SPECT	Improved post-traumatic symptoms (PCL)
Miller et al., 2015 (multicenter, double-blind, sham-controlled clinical trial, HOPPS trial)	47/72	100%	+	1.5 ATA	40	60	1.2 ATA or standard care	None	The post-traumatic symptoms score (PCL) improved in both treatment groups, without a difference between the groups
Cifu et al., 2014 (double-blind, randomized, controlled trial)	?/50	100%	+	2 ATA	40	60	10% O_2_ at 2 ATA (equivalent to room air) or 75% O_2_ at 2 ATA (equivalent to 1.5 ATA)	None	Improved post-traumatic symptoms (PCL) score at 100% 2 ATA group
Harch et al., 2012 (safety study)	15/16	100%	+	1.5 ATA	40	60	NA	SPECT	Improved post-traumatic symptoms (PCL)
Wolf et al., 2012 (single-center, double-blind, randomized, sham-controlled, prospective trial)	?/60	100%	+	2.4 ATA	30	90	1.3 ATA	None	The PTSD symptoms score (PCL) improved in both treatment groups, without a difference between the groups
Harch et al., 2009 (case report)	1/1	100%	+	1.5	39	60	NA	SPECT	Complete resolution of PTSD
Eovaldi and Zanetti, 2010 (case report)	1/1 (acute stress response)	100%	−	2.4 ATA	7	90	NA	None	Complete resolution of post-traumatic symptoms

TBI, traumatic brain injury; ATA, atmosphere absolute; NA, not applicable; PCL, PTSD Checklist; SPECT, single photon emission tomography; CAPS-5, Clinician-Administered PTSD Scale for DSM-5PSS-I, the post-traumatic symptom scale interview.

Early clinical evidence for the potential use of HBOT in humans with PTSD came from case reports. These described significant improvements in PCS and PTSD symptoms following HBOT (Harch et al., 2009; Eovaldi and Zanetti, 2010). A number of pilot studies involving military personnel with prolonged PCS or TBI were conducted after the publication of these case reports, and demonstrated significant improvements in PTSD symptoms after HBOT sessions (Neubauer and James, 1998; Rockswold et al., 2007).

A pilot trial by Harch et al. (2012) included 16 military persons with prolonged PCS due to mild-moderate TBI or blast injury. Fifteen of them were also diagnosed with PTSD. Forty 60-min HBOT sessions of 1.5 ATA were prescribed. Following HBOT, PTSD symptoms improved significantly, as reflected by the decrease in the mean PTSD Checklist-Military (PCL-M), from 67.4 ± 10.5 to 47.1 ± 16 (*P* < 0.001).

A single-center, double-blind, randomized, sham-controlled, randomized is always prospective trial (Wolf et al., 2012) at the U.S. Air Force School of Aerospace Medicine evaluated the effect of 2.4 ATA HBOT vs. room air at 1.3 ATA (prescribed as sham) on post-concussion and post-traumatic symptoms. Fifty military service members with a history of TBI and post-concussion symptoms received 30 sessions of one of the treatments over an 8-week period. Post-traumatic symptoms were evaluated using PCL-M. The article does not mention the number of participants who were diagnosed with PTSD. However, following both the 2.4 ATA and 1.3 ATA protocols, PCL-M improved significantly. Cognitive scores and post-concussion symptoms also improved in both groups.

Miller et al. (2015) recruited 72 veterans with PCS to a multicenter, double-blind, sham-controlled clinical trial. The participants were randomized 1:1:1 to 40 HBOT sessions administered at 1.5 ATA with 100% oxygen, 40 HBOT sessions administered at 1.2 ATA with room air, or no supplemental chamber procedures (standard care). At baseline, 66% of the participants met the criteria for PTSD. Following the intervention, the PTSD symptoms improved in the two active arms (the mean changes of PTSD Checklist score were 11.4; 95% CI, 5.9 to 16.9 and 5.0; 95% CI, −1.7 to 11.6, respectively).

Notably, the use of a hyperbaric environment as a sham treatment aims to enable blinding of group allocation in HBOT trials. However, even a slight increase in oxygen and/or pressure can have meaningful physiological effects, that invalidate the sham condition as a true placebo control. It is well known that any increase in atmospheric pressure, even without changing the concentration, increases gas solubility (Henry’s law). For example, 1.05 ATA, the pressure at the Dead Sea, Israel (−436 m below sea-level), can yield significant physiological effects (Kramer et al., 1998; Abinader et al., 1999). Furthermore, among healthy volunteers, stem cell progenitors were shown to increase by threefold following 10 sessions of 1.2 ATA with 21% oxygen (MacLaughlin et al., 2023). Thus, improvement beyond expectation following sham treatment in a hyperbaric environment suggests that such condition is mistakenly regarded as sham. Accordingly, 1.3 ATA may well serve as a low dosage active treatment rather than as a sham control. Several studies addressed this issue by utilizing alternative methods to provide a placebo-like control condition (Hadanny et al., 2022; Zilberman-Itskovich et al., 2022).

Cifu et al. (2014) recruited 61 veterans with PCS to a double-blind controlled study in which 40 sessions of 2 ATA HBOT were prescribed with 10, 75, or 100% oxygen. The different protocols aimed to enable participants blinding to the group allocation and to serve as equivalents to the common 1.5 ATA 100% protocol, the 2 ATA protocol, and to room air. While PCS did not improve significantly in any of the groups, the PCL-M score decreased from 49.4 at baseline to 42.6 (*P* < 0.05) after treatment in the 2 ATA 100% oxygen group.

In another prospective case-control study, Harch et al. (2017) recruited 30 active service members or veterans with PCS, with or without PTSD. Upon recruitment, 10 of them had symptoms that correlated with the diagnosis of PTSD. After 40, 60-min HBOT sessions, PTSD symptoms improved, as reflected by a decrease in PCL-M, from 63.4 + 15.9 to 46.8 + 16.5 (*P* < 0.001). Continued symptom improvement was observed at 6 months follow-up.

In a retrospective study, Mozayeni et al. (2019) evaluated the effect of 40–82 1.5 ATA HBOT sessions, on neurocognitive test measures, among 32 persons with PCS due to mild TBI. Seven persons (22%) had a diagnosis of PTSD in addition to post-concussive symptoms. Compared to the patients without PTSD, those with a diagnosis of PTSD showed more improvement in fatigue and mood scales (mean change = −23.8 ± 25.1, CI: −32.9 to −14.7, *P* = 0.012), and in neurocognitive test scores (mean change = 13 ± 31, CI: 2–25, *P* = 0.028). Notably, a longer HBOT course was associated with better treatment response.

BIMA (Deru et al., 2018) was another randomized, double-blind, sham-controlled trial of HBOT, for military personnel with post-concussive symptoms, 3 months to 5 years after mild TBI. Forty daily 1-h sessions were provided, with either 100% oxygen at 1.5 ATA or air at 1.2 ATA. Seventy-one patients were randomized, of whom 35 had PTSD. At 13 weeks, the participants who received HBOT showed improvement in post-concussive and PTSD symptoms, sleep quality, control of anger, and memory outcomes, compared to the sham group. Some of the improvements demonstrated after HBOT were greater among the participants with PTSD than among participants with only PCS.

Post-traumatic stress disorder was not the primary recruiting criteria in any of the trials described above; rather, recruitment was according to PCS. PCS and PTSD frequently co-occur (Taylor et al., 2012), as TBI is a strong predictor of PTSD (Chen et al., 2014). Some symptoms of PCS such as fatigue, irritability, sleep disturbances, and concentration difficulties are also common in PTSD. Depression and emotional alterations also frequently occur in both conditions. Thus, differentiating the effect of HBOT on PCS from the effect on PTSD may be challenging.

The first randomized controlled study (Doenyas-Barak et al., 2022) that aimed to evaluate the effect of HBOT on veterans with combat-related PTSD without TBI was published in 2022. The study included veterans who were diagnosed with combat-associated PTSD according to the Israeli Ministry of Defense criteria, and who failed to improve after at least one line of psychological, and or pharmacological treatment. In addition to meeting Ministry of Defense criteria, each participant was evaluated at the time of recruitment by a psychiatrist with expertise in the field of trauma, who validated the diagnosis based on the Diagnostic and Statistical Manual of Mental Disorders 5 (DSM-5) criteria. Individuals were excluded from the trial if they had a history of TBI, or any other brain pathology.

Thirty-five veterans were randomized to HBOT (*N* = 18) or control (*n* = 17) groups; of them, 14 and 15, respectively, completed the protocol. Following HBOT, clinical symptoms improved significantly, according to the Clinician-Administered PTSD Scale for DSM-5 (CAPS-5) inventory, while no change was demonstrated in the control group. Improved brain activity was seen in functional MRI in the left dorsolateral prefrontal, middle temporal gyri, both thalami, left hippocampus, and left insula. The DTI showed a significant increase in fractional anisotropy in the fronto-limbic tracts, genu of the corpus callosum, and fornix.

Long-term follow-up (Doenyas-Barak et al., 2023), performed 704 ± 230 days after completion of the HBOT course, demonstrated persistence of the treatment results. The mean CAPS-5 score (26.6 ± 14.4) was significantly lower than at the pre-HBOT evaluation, 47.5 ± 13.1, *P* < 0.001; and not statistically different from the short-term post-HBOT evaluation, 28.6 ± 16.7, *P* = 0.745. Moreover, for the CAPS-5 subcategory D (cognition and mood symptoms), the mean score was significantly lower at the long-term than short-term evaluation, 7.6 ± 5.1 vs. 10.0 ± 6.0, *P* < 0.001. At the long-term compared to the pre-treatment evaluation, higher percentages of the participants were living with life partners [77% (*n* = 17) vs. 46% (*n* = 10), *P* = 0.011] and were working [73% (*n* = 16) vs. 41% (*n* = 9), *P* = 0.033]. Improvements in long-term follow-up were also consistent with medication use; markedly, the number of benzodiazepine users decreased, from 10 (46%) to 4 (18%) (*P* = 0.07), and the median medically prescribed cannabis dose decreased from a monthly 40.0 g (0–50) to 22.5 g (0–30) per month (*P* = 0.046). The long-term beneficial effect, more than 2 years after the last HBOT session, further supported the regenerative effect of HBOT. Unlike pharmacotherapy, which obligates permanent administration, or intensive psychotherapy, whose effects do not persist after treatment cessation (Jaycox and Foa, 1996; Hertzberg et al., 2002; Rapaport et al., 2002; Davidson, 2004), the biological benefit of HBOT persisted for years. These results suggest that the regenerative effects induced by HBOT promote lasting tissue repair and a new biological equilibrium.

As HBOT obligates daily arrival at the hyperbaric center, and the expectation from the treatment is high, a potential placebo effect should be considered. Thus, a second, placebo-controlled trial with similar combat-related PTSD population is currently being conducted by the Sagol center research group. To annul hyperbaric conditions, but provide pressure sensation to the ears, the pressure in the placebo condition is increased to 1.1 ATA during the first 5 min of the session, with hissing noise of circulating air. The pressure is then decreased slowly during the next half hour, to 1.0 ATA, with an oxygen level of 21%. Positive outcomes of this study may contribute to validation of the effect of HBOT on PTSD.

## HBOT for PTSD induced by childhood sexual abuse

An effect of HBOT was suggested among individuals with PTSD induced by childhood sexual abuse. A prospective randomized controlled clinical trial by Hadanny et al. (2018b) included 30 women with fibromyalgia and a history of childhood sexual abuse. Following 60 HBOT sessions at 2 ATA, entailing 100% oxygen for 90 min with 5-min air breaks every 20 min, significant improvement was observed in fibromyalgia- and PTSD-related symptoms. PTSD-related symptoms, such as somatization, depression, and anxiety were correlated with improvements in metabolic brain activity, as assessed by brain SPECT.

## Adverse effects

Hyperbaric oxygen therapy is generally safe and well tolerated. The vast majority of side effects are mild and reversible (Camporesi and Bosco, 2014). Middle ear barotrauma is the most common side effect of hyperbaric oxygen, with an incidence of about 2% (Camporesi and Bosco, 2014), and a slightly higher frequency among those who undergo multiple treatments (Bessereau et al., 2010). Sinus barotrauma is another reversible common complication of hyperbaric oxygen, and usually presents in patients with upper respiratory tract infections or allergic rhinitis (Camporesi and Bosco, 2014).

Some patients present with reversible myopia due to direct oxygen toxicity to the lens. While its etiology is unclear, it usually resolves within days to weeks after the last treatment (Camporesi and Bosco, 2014).

Pulmonary barotrauma is an unusual side effect of HBOT, provided that pneumothorax was excluded before initiating HBO therapy (Leach et al., 1998). Other pulmonary adverse effects such as pulmonary edema, chest tightness, and cough have rarely been reported in conjunction with HBOT (Fan et al., 2017).

Seizures due to central nervous system oxygen toxicity are a rare but dramatic consequence of HBOT (Hadanny et al., 2016; Manning, 2016). Patients receiving glucocorticoids, insulin, thyroid replacement, and sympathomimetic medications may be at higher risk of oxygen toxicity of the central nervous system.

Hyperbaric oxygen therapy has also been associated with hypoglycemia in some individuals with diabetes (Roth and Weiss, 1994). A retrospective analysis reported adverse events among 406 (17.4%) of 2,334 patients who underwent an HBOT course; the overall incidence was 721:100,000 events per session (0.72%) (Hadanny et al., 2016). Subjective symptoms of barotraumas [otalgia (Hadanny et al., 2016), sinus pain] were reported by 79 (3.4%) individuals, while 215 (0.36%) had objective signs of middle ear barotrauma per otoscopy and 16 (0.02%) had objective sinus barotrauma. Only one individual had a HBOT related seizure. A total of 58 (2.5%) individuals did not complete the prescribed HBOT sessions due to side effects. The main reason for treatment termination was middle ear barotrauma (55%).

## Challenges specific to treating PTSD with HBOT

Individuals with PTSD have often reported worsening of symptoms during the HBOT course. Harch et al. (2012) reported temporary worsening of emotional lability, depression, and headache in four of 16 recruited persons. Miller et al. (2015) reported worsening claustrophobia in one person. Among individuals who were recruited to a study on fibromyalgia related to child abuse, fibromyalgia symptoms worsened temporarily, at about the 20th session in most (Hadanny et al., 2018b). As the HBOT course progressed, symptoms resolved in all the patients. By the end of the HBOT treatment, clinical improvement was significant compared to baseline pre-HBOT assessments.

Among persons with fibromyalgia (Efrati et al., 2018), a unique phenomenon of memory recollection was first reported. Similarly, recollection of inaccessible memories was reported in 35.7% of veterans with military-related PTSD (Doenyas-Barak et al., in press). The memories surfaced mostly during the second month of the treatment; and their recollection was accompanied by temporary worsening of PTSD symptoms, and/or by somatic pain. Most of the reported resurfaced memories were related to traumatic events; nevertheless, it is important to note that changes in access to non-traumatic memories cannot be ruled out. Furthermore, in the majority of cases, the accuracy of these resurfaced memories could not be verified. While memory recollection and the accompanied distress may be considered adverse effects of the treatment, they may also represent an “on target” effect that contributes to hippocampal-based memory processing in individuals with PTSD.

Rare cases of hypomania associated with HBOT have raised concern regarding the safety of this treatment for individuals with a history of psychosis related to schizophrenia or bipolar disorder (unpublished clinical data). While further research of these issues is currently underway, we exclude persons with co-occurring PTSD and recent or frequent psychosis from HBOT. Further, the potential recollection of inaccessible traumatic memories and the potential for worsening of PTSD-related symptoms during the course of HBOT treatment, emphasizes the need for dedicated professional medical staff with expertise in PTSD and HBOT. Additionally, given that some cases of memory recollection have been reported in patients without a known history of traumatic experiences (Efrati et al., 2018), and considering that bipolar disorder or psychosis can occur in non-traumatized populations, it is important that all medical professionals in each HBOT center be trained in handling such cases.

## Discussion

Pre-clinical and clinical trials have shown that HBOT can induce neuroplasticity and improve clinical outcomes of veterans with treatment-resistant PTSD. The biological effects of HBOT include improved mitochondrial function, stem cell proliferation, angiogenesis, and neurogenesis. A number of case reports and 10 clinical trials, including six controlled trials, evaluated the effects of HBOT on PTSD. All those studies indicated positive effects on PTSD symptoms. As detailed above, particular attention should be given to the methods used in the trials, the treatment protocol, the duration of HBOT sessions, and the handling of the control group. In the various studies, treatment pressures ranging from 1.5 ATA to 2.4 ATA were prescribed, demonstrating a high safety profile and significant effects on PTSD symptoms when evaluated shortly after treatment completion. In four of the mentioned trials (Wolf et al., 2012; Cifu et al., 2014; Miller et al., 2015; Deru et al., 2018), the control groups were treated with lower doses of HBOT, which were shown to have some biological effects.

The number of sessions varied among the studies, ranging from 30 to 80 sessions. Although the different protocols were all proven to be safe, the induction of neuroplasticity requires long treatment courses. In our center, we prescribe 60 daily sessions, given 5 days per week. However, the exact minimal effective dosage has not been determined and further research is needed.

Most military-related clinical trials focused on evaluating the effects of HBOT on PCS, whereby PTSD was a common comorbidity and was also assessed. Therefore, the improvements in post-traumatic symptoms observed in these trials may be partially attributed to the alleviation of post-concussion symptoms. Thus far, two clinical trials specifically excluded individuals with a history of physical trauma (Hadanny et al., 2018b; Doenyas-Barak et al., 2022), allowing for a clearer assessment of the effects of HBOT on PTSD symptoms. Both trials demonstrated significant improvement in clinical outcomes.

Taken together, HBOT presents a novel therapeutic approach for PTSD, that targets the biological consequences of traumatic events; by inducing a cascade of salutary physiological alterations that culminate in regenerative neuroplasticity, it offers clinical relief to many who had been suffering from long-term, persistent symptoms of PTSD.

As over many years, HBOT has been used in clinical practice for various indications, such as non-healing peripheral ischemic wound, its safety profile is known. When administered by a trained professional medical team and when using medical standard hyperbaric chambers, HBOT is considered safe and the potential side effects are typically self-remitting. However, certain aspects are specifically relevant for individuals with PTSD, and these need particular attention. During the HBOT treatment course, recollection of traumatic memories may occur. Surfacing of inaccessible memories were reported to occur in 35.7% of individuals with military-related PTSD (Doenyas-Barak et al., in press). The new memories surfaced mostly during the second month of the treatment, and the surfacing was accompanied by temporary worsening of PTSD symptoms. The distress resolved gradually, during the course of a few days, and the memory could be integrated into the participants’ narratives. It is highly important that any medical team that treats patients with PTSD by HBOT be aware of this phenomenon and know how to address it. In addition, based on our center’s experience, we do not recommend exposure therapy during HBOT sessions. The distress associated with exposure techniques could potentially hinder progress. Instead, we believe that strengthening self-regulatory techniques may contribute to a safer treatment process and support neuroplasticity. Therefore, collaboration with the treating psychologist is necessary to ensure appropriate supplementary treatment.

While preclinical data contribute to our understanding of the potential mechanisms underlying the beneficial effects of HBOT on PTSD, further clinical trials are needed to assess their role in patients with PTSD. The utilization of biomarkers in future trials may help optimize and individualize the HBOT protocol. The relatively new use of functional imaging of the brain, that is being evaluated may also hold promise for individualizing the HBOT protocol per patient pathology. More research and clinical experience are also needed with regard to the accompanying treatments and interventions that may further enhance the clinical benefit gained by HBOT.

## Author contributions

KD-B: Conceptualization, Investigation, Writing – original draft. IK: Investigation, Writing – review and editing. EL: Investigation, Writing – review and editing. RM: Writing – review and editing. RL: Writing – review and editing. RB-G: Writing – review and editing. SE: Conceptualization, Supervision, Writing – review and editing.
